# Retinal Sensitivity before and after Silicone Oil Removal Using Microperimetry

**DOI:** 10.1155/2019/2723491

**Published:** 2019-04-11

**Authors:** Ghada A. Nassar, Maha M. Youssef, Lameece M. Hassan, Hebatalla S. Makled

**Affiliations:** Department of Ophthalmology, Cairo University, Faculty of Medicine, Cairo 11562, Egypt

## Abstract

The purpose of the study is to assess the retinal sensitivity, using microperimetry, before and after silicone removal. It included 22 patients admitted for silicone removal after vitrectomy for macula-off retinal detachment. Patients were divided into 2 groups according to the duration of silicone tamponade: Group A: <3 months (included 10 patients), and Group B: 3–6 months (included 12 patients). Retinal sensitivity was tested, using microperimetry, one day before and one month after silicone removal. The best-corrected visual acuity (in LogMAR) significantly improved postoperatively (0.69 versus 1.06 and 0.69 versus 1.07 in Groups A and B, respectively). The mean intraocular pressure (IOP) was 12.89 ± 1.05 mmHg postoperatively versus 14.89 ± 1.76 mmHg preoperatively in Group A (*p*=0.011) and was 13.33 ± 1.30 mmHg postoperatively versus 15.33 ± 3.11 mmHg preoperatively in Group B (*p*=0.008). In Group A, the mean postoperative overall retinal sensitivity was 8.70 ± 2.56 dB versus 5.68 ± 2.00 dB preoperatively (*p*=0.008). In Group B, it was 9.83 ± 3.36 dB versus 7.00 ± 2.55 dB (*p*=0.002). No statistically significant difference was found between the two groups as regards improvement in overall retinal sensitivity. We concluded that the overall retinal sensitivity significantly increased following silicone removal in both groups. This trial is registered with ISRCTN43187564.

## 1. Introduction

Silicone oil is a widely used tamponading agent in vitreoretinal surgery, especially in cases of complicated retinal detachment [1]. However, it is often associated with complications such as cataracts, glaucoma, and corneal decompensation [2, 3]. Moreover, silicone oil is potentially retinotoxic [4, 5].

Visual loss associated with silicone use has been reported either during the period of tamponade or suddenly at the time of its removal [4, 6, 7]. Since the visual loss is often unexplained, it may be attributed to the possible toxic effect of silicone on the retina [4].

Microperimetry is a field test that allows simultaneous fundus visualization, thus allowing correlation between functional defects and underlying morphological changes [8]. It has been used to detect the retinal toxicity in many conditions such as in hydroxychloroquine therapy [9].

The aim of this work is investigating the effect of silicone oil on the retina through assessment of retinal sensitivity, using microperimetry, before and after silicone removal. As a secondary outcome, the change in overall retinal sensitivity will be correlated with change in the best-corrected visual acuity (BCVA).

## 2. Materials and Methods

This prospective comparative study took place between November 2016 and May 2017 and included 22 eyes of 22 patients admitted in the Department of Ophthalmology, Cairo University, for silicone oil removal. It was approved by our ophthalmology department, and a written informed consent was obtained from all patients before enrollment.

All patients underwent 23-guage pars plana vitrectomy with silicone oil (BIOSIL silicone oil 5000 centistokes by OMNIA Fluid, Italy) injection for rhegmatogenous macula-off retinal detachment.

Patients undergoing vitrectomies for proliferative diabetic retinopathy, recurrent retinal detachments, or macular holes, were excluded from the study. Patients with chorioretinal degenerations involving the macula and those who developed complications in the form of visually significant cataract, secondary glaucoma with intraocular pressure (IOP) exceeding 26 mmHg, silicone oil emulsification, recurrent detachment, or hypotony following oil removal were also excluded.

The preoperative data including age, gender, and medical history were recorded for all patients. Three days before and one month after silicone oil removal, all patients underwent full ophthalmological examination in the form of measurement of BCVA using Snellen charts and then converted into logMAR for statistical analysis, IOP measurement using Goldmann applanation tonometry, slit-lamp examination, and dilated fundus examination. Patients were divided into 2 groups according to the duration of silicone tamponade: Group A: silicone tamponade less than 3 months, and Group B: silicone tamponade from 3–6 months.

### 2.1. Surgery Technique

The two-port (infusion-extraction) technique was adopted for silicone oil removal, and two standard sclerotomies were made over the temporal pars plana. One was fitted with a standard infusion line connected to the bottle containing BSS, and silicone oil was extracted through the second sclerotomy using a 20-gauge silicone injection/aspiration cannula.

The retinal sensitivity was evaluated by microperimetry using the OPTOS Spectral OCT/SLO (scanning laser ophthalmoscope) Combination Imaging system (OPTOS, Inc., FL, USA).

### 2.2. Microperimetry Technique

The microperimetry examination was performed after explanation of the technique to all patients. To exclude the learning effect, patients were examined 2 days before silicone oil removal and results of the first test were discarded. The second measurement was performed on the next day, and the third measurement one month after silicone oil removal. The patients were examined in a dark room for 15 minutes with occlusion of the nontested eye. They were asked to maintain fixation on a central target. A customized pattern centered on the central 11° was used, after autocorrection of patient's refractive error by the machine, with the following features: Goldmann III stimulus size, 200 millisecond stimulus duration, a 1,500 millisecond interval between stimuli, and a 4–2 strategy on a 1.27 cd/m^2^ background.

The retinal sensitivity was tested at 28 points: 4 stimuli at 2.3°, 12 stimuli at 6.6°, and 12 stimuli at 11°. The stimulus level ranged between 0 dB and 20 dB. The total retinal sensitivity and sensitivity of each layer (inner, middle, and outer) were assessed.

### 2.3. Statistical Analysis

Data were coded and entered using the statistical package for the Social Sciences SPSS (IBM, Armonk, NY) version 24. Data were summarized using mean, standard deviation, median, and minimum and maximum in quantitative data and using frequency (count) and relative frequency (percentage) for categorical data. Comparisons between quantitative variables were done using the nonparametric Mann–Whitney test. For comparison of serial measurements within each patient the nonparametric Wilcoxon signed-rank test was used. For comparing categorical data, the chi-squared (*χ*^2^) test was performed. The exact test was used instead when the expected frequency is less than 5. *p* values less than 0.05 were considered as statistically significant.

## 3. Results

Twenty-two eyes of 22 patients that fulfilled the inclusion criteria were identified.

### 3.1. Epidemiology and Clinical Data

The mean age of the patients in Group A was 36.7 ± 10.9 years (range: 19–49), while in Group B, it was 39.4 ± 8.8 years (range: 25–52).

Group A included 3 females and 7 males (10 patients). Group B included 3 females and 9 males (12 patients). As regards the lens status, Group A had 3 phakic patients and 7 pseudophakic patients, while in Group B, all patients were pseudophakic.

The BCVA significantly improved postoperatively in both groups. In Group A, BCVA (logMAR) was 0.69 versus 1.06 (*p*=0.007). Also, it was 0.69 versus 1.07 (*p*=0.002) in Group B.

The IOP was significantly reduced postoperatively. The mean IOP was 12.89 ± 1.05 mmHg postoperatively versus 14.89 ± 1.76 mmHg preoperatively in Group A (*p*=0.011). In Group B, it was 13.33 ± 1.30 mmHg postoperatively versus 15.33 ± 3.11 mmHg preoperatively (*p*=0.008).

### 3.2. Microperimetry Data

The total retinal sensitivity values were significantly increased postoperatively in both groups. In Group A, the mean postoperative total retinal sensitivity was 8.70 ± 2.56 dB versus 5.68 ± 2.00 dB preoperatively (*p*=0.008). Similarly, in Group B, it was (9.83 ± 3.36 dB) postoperatively versus (7.00 ± 2.55 dB) preoperatively (*p*=0.002).

The retinal sensitivity at the inner, middle, and outer rings was significantly improved postoperatively when compared to the preoperative values for both groups. This is shown in Tables 1 and 2.

When comparing the preoperative and the postoperative micro-perimetric values between the two groups, there was no statistically significant difference found between the two groups as regards the improvement in the average total retinal sensitivity, as well as the sensitivity in the inner, middle, and outer rings. This is shown in Table 3.

Improvement in the postoperative BCVA was positively correlated with improvement in postoperative total retinal sensitivity (*r* = 0.060). This correlation did not reach the statistical significance value (*p*=0.796). However, one of the limitations of our study was the small number of patients in each group in order to yield a statistically significant result.

## 4. Discussion

Use of silicone oil (SO) tamponade, although a common practice, may have a deleterious effect on retinal function.

In this study, we investigated the effect of silicone oil on the retina by using microperimetry to assess retinal sensitivity, before and after its removal. It was found that the overall retinal sensitivity, as well as the sensitivity at inner, middle, and outer rings, increased significantly following silicone oil removal in both groups. Thus, the retinal sensitivity improved after silicone oil removal.

However, the duration of the tamponade was not found to significantly affect the retinal sensitivity. There was no statistical significance between the 2 groups as regards the improvement in the overall retinal sensitivity or the sensitivity in the three rings unlike Scheerlinck et al., where the duration of SO tamponade was the only statistically significant factor related to the incidence of unexplained visual loss (*p*=0.001) [10]. On the contrary, no correlation was revealed between retinal thinning and duration of silicone tamponade in the study carried out by Lee and colleagues [11].

Improvement in the postoperative BCVA, in our study, was positively but not significantly correlated with improvement in postoperative total retinal sensitivity (*p*=0.796). In another study comparing the use of silicone oil versus gas tamponade in retinal detachment, the retinal sensitivity on microperimetry did not correlate well with BCVA within individual patients. This was attributed to the varied effect of intraocular SO on retinal functions (resolving spatial patterns for acuity and sensitivity in microperimetry) [12].

The improvement of retinal sensitivity following silicone oil removal remains unexplained. Complications of silicone oil include cataracts, silicone emulsification, and secondary glaucoma, which can negatively affect vision and retinal sensitivity, but could improve after silicone oil removal. However, in our study, we strictly excluded these patients.

Although we excluded patients with secondary glaucoma and the mean preoperative IOP in our study was within normal range, we reported significant decrease in IOP postoperatively. It is known that increased IOP may cause mechanical stress to the fovea, leading to loss of outer nuclear layer cell bodies [13]. Thus, this decrease in IOP (even though it remained within the normal range) could be a possible explanation for improvement in retinal sensitivity. More recently, a study on macula-on retinal detachment found that increased IOP during SO endotamponade was the most important risk factor for visual loss [14].

Several hypotheses have been proposed to explain the pathophysiology of the potential toxic effects of silicone. The dissolution of lipophilic macular pigments in silicone oil may render the macula more susceptible to phototoxicity [4, 15–17]. Another theory is the loss of the buffering capacity of vitreous, leading to impaired homeostasis and potassium accumulating in the retina with silicone tamponade. This may cause degeneration of Müller cells [18]. Moreover, the retro-oil fluid contains elevated levels of cytokines, which may affect retinal function by inducing apoptosis and neuronal degeneration, resulting in retinal thinning [19].

However, it is not well known which of these hypotheses could be reversible, explaining the significant improvement in retinal sensitivity that occurred after silicone oil removal in our study. An earlier study, which reported improvement in the visual function after silicone oil removal, attributed it, in part, due to continued improvement in the retinal function following successful retinal reattachment [20].

Several case reports and series have reported unexpected visual loss in patients with macula-on retinal detachment after vitrectomy and silicone oil injection. This could happen either during silicone oil tamponade [4, 21, 22] or at time of silicone removal [6, 7, 21, 23, 24]. This did not occur in our study and on the contrary vision improved in both groups.

Our study differed from the aforementioned case series, especially with regard to the study design and included patients. Most of them were retrospective in nature, and their cases were macula-on rhegmatogenous retinal detachment (RRD). Our study was prospective and all patients had detached maculae prior to vitrectomy. Moreover, the visual loss described in these series was found to be permanent in most patients, while visual acuity and retinal sensitivity in our study improved in all cases. Electrophysiological studies in these patients did not show consistent changes; however, some evidence of damage to ganglion and bipolar cells in macular area was found [4, 7, 10]. Based on these findings, macular dysfunction is proposed as the most likely cause of silicone oil related visual loss.

In 2018, Scheerlinck et al. proposed that cases with silicone oil related visual loss and macula-off RRD may go unnoticed because their poor vision is already explained by a detached macula. However, against their theory, they did not find any differences in retinal sensitivity between macula-on and macula-off RRD in their study [12].

**Figure 1:**
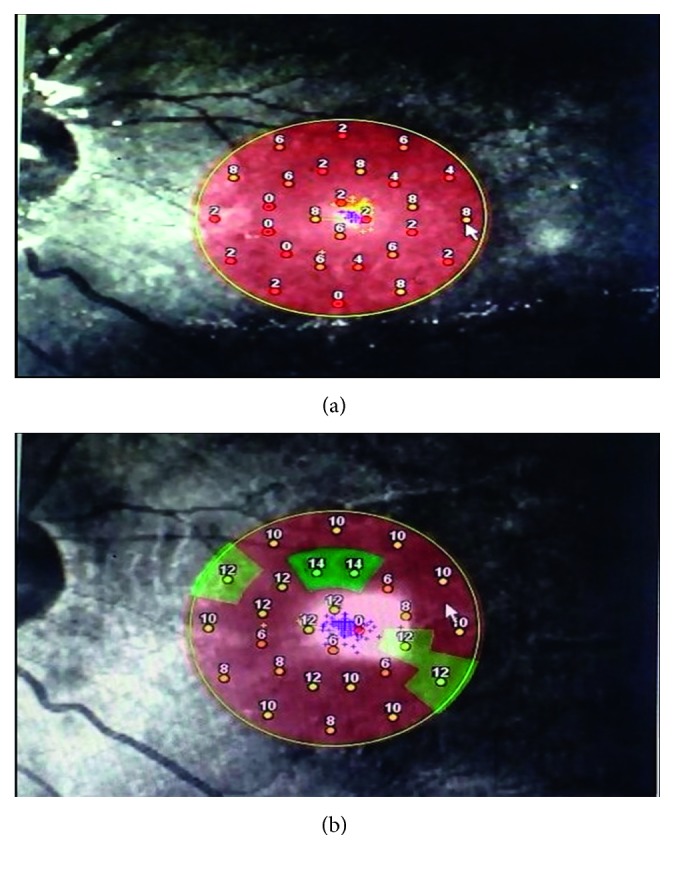
(a, b) Microperimetry polar 3–12° test (patient No. 5, left eye) superimposed on scanning laser ophthalmoscope infrared image. The test points were arranged within the macula showing the sensitivity values in 3 concentric circles centered on the fovea (a) with a preoperative mean retinal sensitivity of (4 dB) and (b) with a postoperative mean retinal sensitivity of (9.6 dB).

However, Scheerlinck and colleagues tested retinal sensitivity, using microperimetry, in 10 out of 20 patients who experienced unexplained visual loss following vitrectomy and silicone oil injection for macula-on detachment (but not preoperatively). A severely reduced central sensitivity on microperimetry was detected in these patients. This central scotoma differed in patients of retinal detachment with macular involvement, whose retinal sensitivity was reduced at all stimuli, as detected in our patients [10], as shown in Figures 1(a) and 1(b).

Macular dysfunction after the use of silicone oil, in patients with unexplained visual loss after vitrectomy for macula-on detachment, has been supported by changes on optical coherence tomography (OCT). Patients showed microcystic edema in the inner nuclear layer and thinning of inner retinal layers [10, 21, 23]. In contrast, a more recent study demonstrated that silicone oil caused significant thinning of each retinal layer, not only the inner retinal layers [11].

Likewise, patients with detached maculae showed central foveal atrophy or thickening after pars planavitrectomy and silicone oil injection [25, 26]. Unfortunately, we have not included OCT in our study, which would have allowed the correlation between functional and structural evaluation of macula.

The main strength of this study is that it is the first to use microperimetry to compare retinal sensitivity in the same patients prior to and after silicone oil removal. Since all secondary complications were excluded, we can conclude that the improvement was most likely related to the removal of the silicone oil. However, one of the limitations of our study was the small number of patients in each group in order to yield a statistically significant result.

## 5. Conclusion

In conclusion, with the use of microperimetry, we noticed significant improvement of retinal sensitivity following silicone oil removal. However, the pathogenesis of silicone oil toxicity remains unclear.

Larger prospective studies are required for better understanding the potential toxic effect of silicone oil on the retina and determining the proper timing of silicone oil removal. Longer postoperative follow-up may allow us to determine the degree of recovery from silicone oil toxicity. These studies should combine both functional and structural evaluation of macula. Microperimetry will remain a valuable diagnostic tool in such studies.

## Figures and Tables

**Table 1:** The preoperative and postoperative values of the inner, middle, and outer rings of the retinal sensitivity at microperimetry in Group A.

	Mean	SD	*p* value
Pre-inner RS (dB)	4.63	1.76	0.012
Post-inner RS (dB)	6.97	2.25	
Pre-middle RS (dB)	4.80	2.33	0.008
Post-middle RS (dB)	7.57	2.67	
Pre-outer RS (dB)	6.30	2.34	0.008
Post-outer RS (dB)	8.94	2.72	

SD: standard deviation; RS: retinal sensitivity.

**Table 2:** The preoperative and postoperative values of the inner, middle, and outer rings of the retinal sensitivity at microperimetry in Group B.

	Mean	SD	*p* value
Pre-inner RS (dB)	6.85	3.81	0.005
Post-inner RS (dB)	9.60	3.14	
Pre-middle RS (dB)	6.65	2.81	0.003
Post-middle RS (dB)	8.95	3.42	
Pre-outer RS (dB)	7.69	2.46	0.002
Post-outer RS (dB)	10.39	3.48	

SD: standard deviation; RS: retinal sensitivity.

**Table 3:** The values of improvement in the average total retinal sensitivity as well as sensitivity in the inner, middle, and outer rings in Group A and Group B.

	Mean in Group A (dB)	Mean in Group B (dB)	*P* value
Improvement in the total RS	3.02	2.83	0.569
Improvement in the inner RS	2.33	2.75	0.972
Improvement in the middle RS	2.77	2.30	0.499
Improvement in the outer RS	2.64	2.70	0.803

RS: retinal sensitivity.

## Data Availability

All the data used and/or analysed during the current study are available and can be presented by the corresponding author upon a reasonable request.
